# Supplementary material for: RNA-Seq Analysis of Cisplatin and the Monofunctional Platinum(II) Complex, Phenanthriplatin, in A549 Non-Small Cell Lung Cancer and IMR90 Lung Fibroblast Cell Lines
Source: Cells. 2020 Dec 8;9(12):2637. doi: 10.3390/cells9122637 (PMC7764052; doi:10.3390/cells9122637)
Supplement: Supplementary file 1 [file cells-09-02637-s001.pdf]

Supplementary Table 1: Sample and Barcode Information

Sample_ID	I7 Index ID	I7 Index
MS_01_A549_untreated_vehicle_1	BC001	CGCTACAT
MS_02_A549_untreated_vehicle_2	BC002	AATCCAGC
MS_03_A549_phenanthriplatin_1	BC003	CGTCTAAC
MS_04_A549_phenanthriplatin_2	BC004	AACTCGGA
MS_06_A549_cisplatin_1	BC006	ACAACAGC
MS_07_IMR90_untreated_vehicle_1	BC007	ATGACAGG
MS_08_IMR90_untreated_vehicle_2	BC008	GCACACAA
MS_09_IMR90_phenanthriplatin_1	BC009	CTCCTAGT
MS_10_IMR90_phenanthriplatin_2	BC010	TCTTCGAC
MS_12_IMR90_cisplatin_1	BC012	ACTCCTAC
MS_13_A549_untreated_vehicle_3	BC013	CTTCCTTC
MS_14_A549_phenanthriplatin_3	BC014	ACCATCCT
MS_17_A549_cisplatin_2	BC017	GTACACCT
MS_18_A549_cisplatin_3	BC018	ACGAGAAC
MS_19_IMR90_untreated_vehicle_3	BC019	CGACCTAA
MS_20_IMR90_phenanthriplatin_3	BC020	TACATCGG
MS_23_IMR90_cisplatin_2	BC023	TCTAGGAG
MS_24_IMR90_cisplatin_3	BC024	CTCGAACA

Supplementary Table 2: Summary of initial sequence analysis

File	Input Reads	Aligned Reads	Alignment Rate
MS_01_A549_untreated_vehicle_1-288005557	29,624,735	29,443,904	99.38%
MS_02_A549_untreated_vehicle_2-288012171	24,354,246	24,195,940	99.34%
MS_13_A549_untreated_vehicle_3-288019954	27,373,112	27,130,180	99.11%
MS_03_A549_phenanthriplatin_1-288021973	27,068,350	26,848,365	99.18%
MS_04_A549_phenanthriplatin_2-288029943	26,167,494	25,935,865	99.11%
MS_14_A549_phenanthriplatin_3-288021975	25,804,338	25,557,744	99.04%
MS_06_A549_cisplatin_1-288029944	25,993,579	25,803,612	99.26%
MS_17_A549_cisplatin_2-288026940	26,612,411	26,457,257	99.41%
MS_18_A549_cisplatin_3-288010192	28,711,302	28,530,222	99.36%
MS_07_IMR90_untreated_vehicle_1-288020948	30,233,132	30,061,035	99.43%
MS_08_IMR90_untreated_vehicle_2-288010190	28,535,621	28,343,931	99.32%
MS_19_IMR90_untreated_vehicle_3-288013976	29,342,292	29,157,183	99.36%

MS_09_IMR90_phenanthriplatin_1-288019952	25,186,144	25,019,000	99.33%
MS_10_IMR90_phenanthriplatin_2-288018959	25,835,999	25,664,633	99.33%
MS_20_IMR90_phenanthriplatin_3-288024879	27,500,038	27,326,280	99.36%
MS_12_IMR90_cisplatin_1-288013974	27,263,332	27,088,420	99.35%
MS_23_IMR90_cisplatin_2-288026945	25,764,318	25,618,335	99.43%
MS_24_IMR90_cisplatin_3-288022955	27,726,156	27,533,265	99.3%

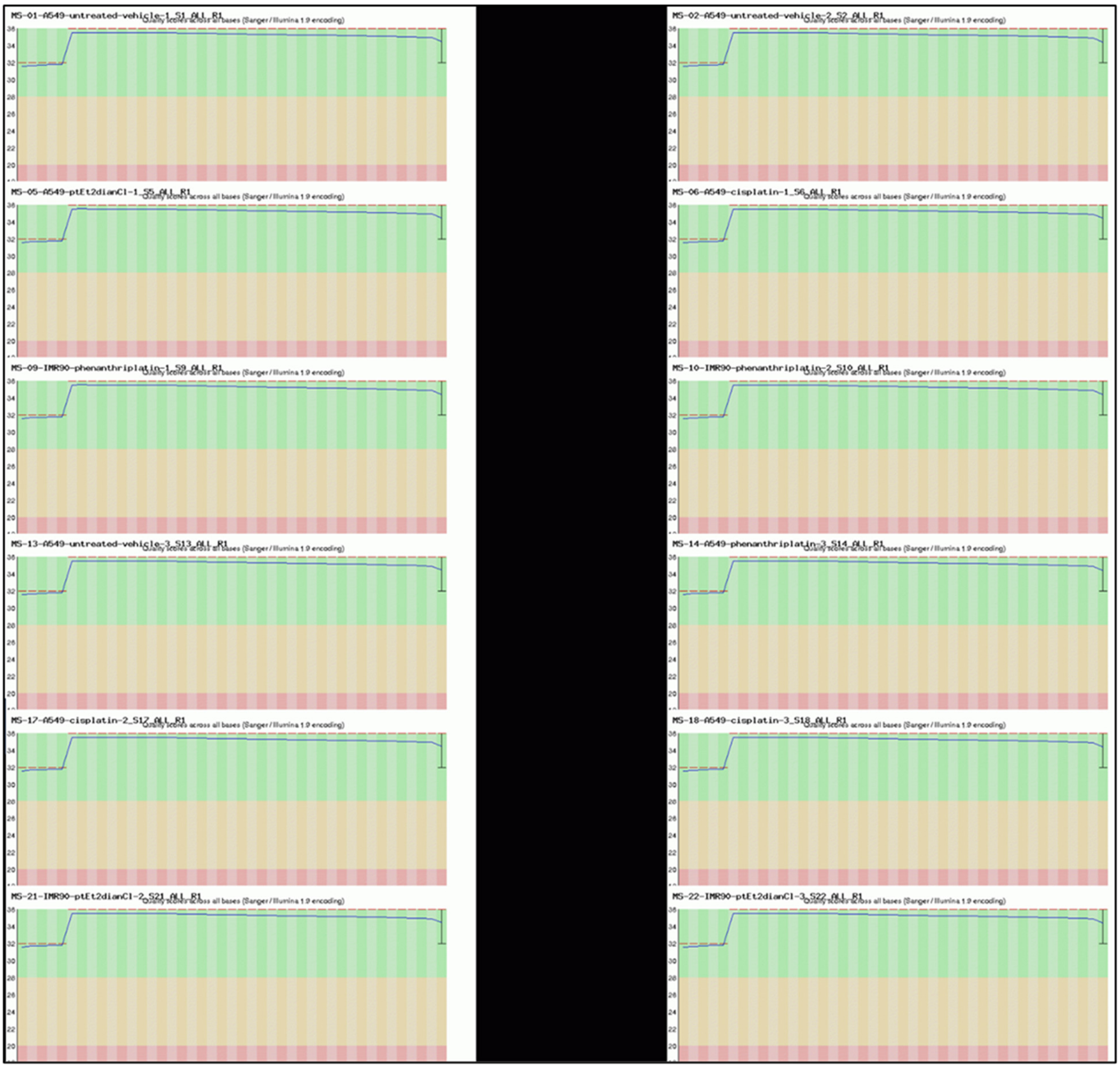

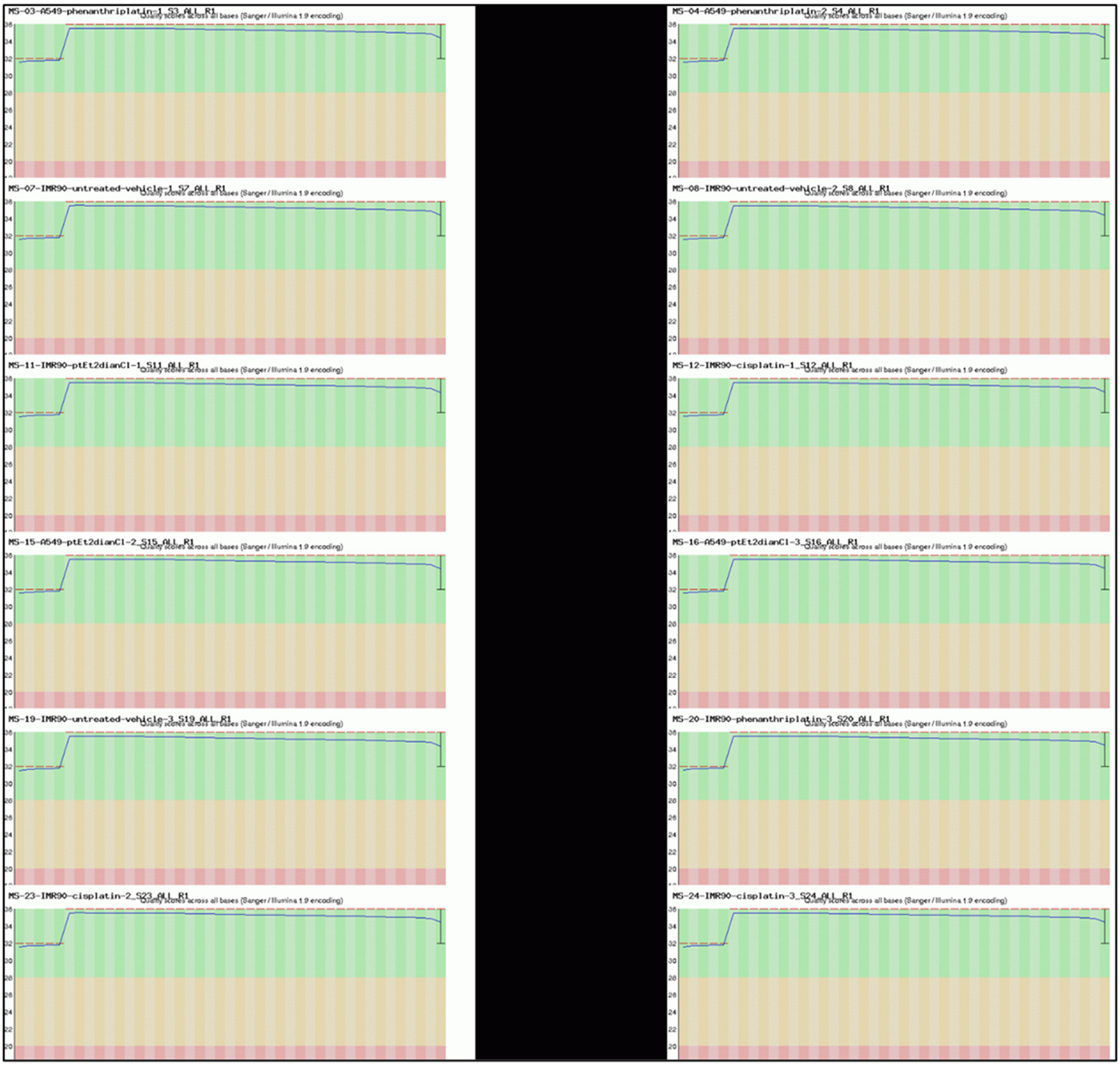

Supplementary Figure 1: Quality scores for raw sequences. The X-axis represents each base position along the reads. The Y-axis represents Phred scores, a measure of the accuracy in base calling. A Phred score of 30 indicates 99.9% accuracy in base calling. Phred scores above 28 (green) are considered very good quality. Scores between 20 and 28 (orange) are considered reasonable quality. Scores below 20 (red) are considered poor quality. The yellow box represents the inter-quartile range (25-75%). The lower and upper whiskers represent the 10th and 90th percentiles respectively.

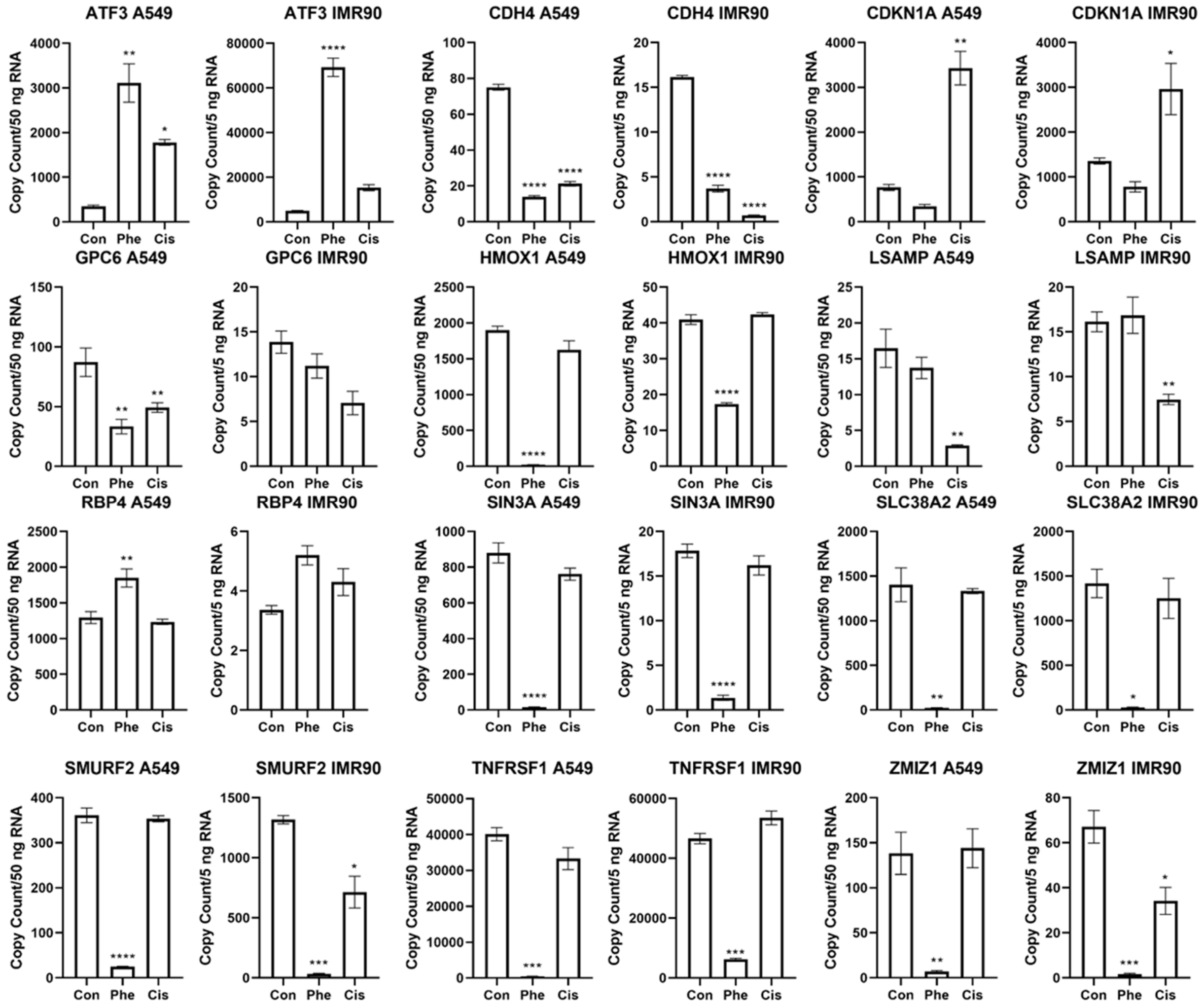

Supplementary Figure 2: Additional examples of next generation sequencing validation using droplet digital polymerase chain (ddPCR) reaction in A549 and IMR90 cells. Mean expression of the experimental genes, ATF3, CDH4, CDKN1A, GPC6, HMOX1, LSAMP, RBP4, SIN3A, SLC38A2, SMURF2, TNFRSF1, ZMIZ1, as measured by ddPCR. Graph labelling: Con = control; Phe = phenanthriplatin; Cis = cisplatin. Y-axis represents copy count for 50 ng RNA sample. Mean (± SEM); N = 3; $p < 0.05$; "*" = $p < 0.05$; "**" = $p < 0.01$; "***" = $p < 0.001$; "****" = $p < 0.0001$.
